# Supplementary figures and images for: Longitudinal variations in the gastrointestinal microbiome of the white shrimp, Litopenaeus vannamei
Source: PeerJ. 2021 Aug 2;9:e11827. doi: 10.7717/peerj.11827 (PMC8340905; doi:10.7717/peerj.11827)

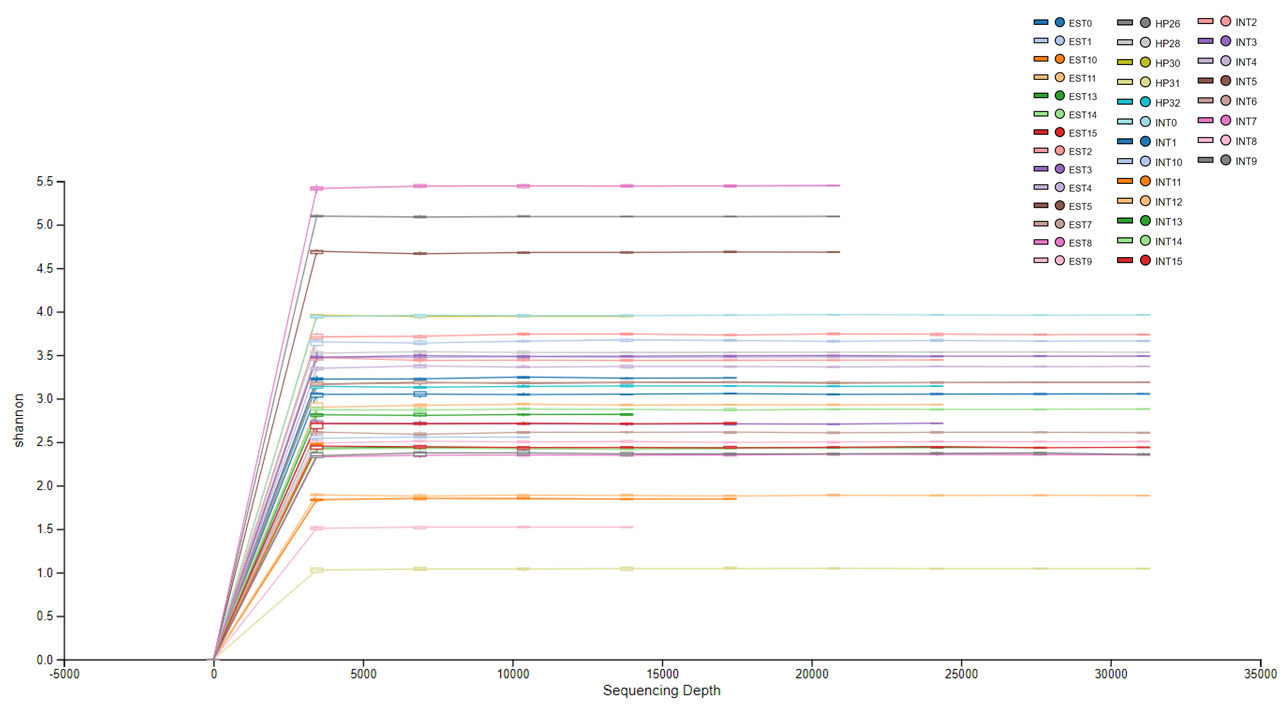


**Figure S1.** Rarefaction curves constructed based on observed ASVs.

Supplement: Supplemental Information 1 [file peerj-09-11827-s001.docx]
