# Supplementary material for: Longitudinal variations in the gastrointestinal microbiome of the white shrimp, Litopenaeus vannamei
Source: PeerJ. 2021 Aug 2;9:e11827. doi: 10.7717/peerj.11827 (PMC8340905; doi:10.7717/peerj.11827)
Supplement: Supplemental Information 2 — Proteobacteria (0.643), Actinobacteria (0.743), Verrucomicrobia (0.767), Firmicutes (0.614), Bacteroidetes (0.831), Cyanobacteria (0.983). Low taxa are the group of taxa with an abundance lower than 1% of the total abundance. [file peerj-09-11827-s002.docx]

**
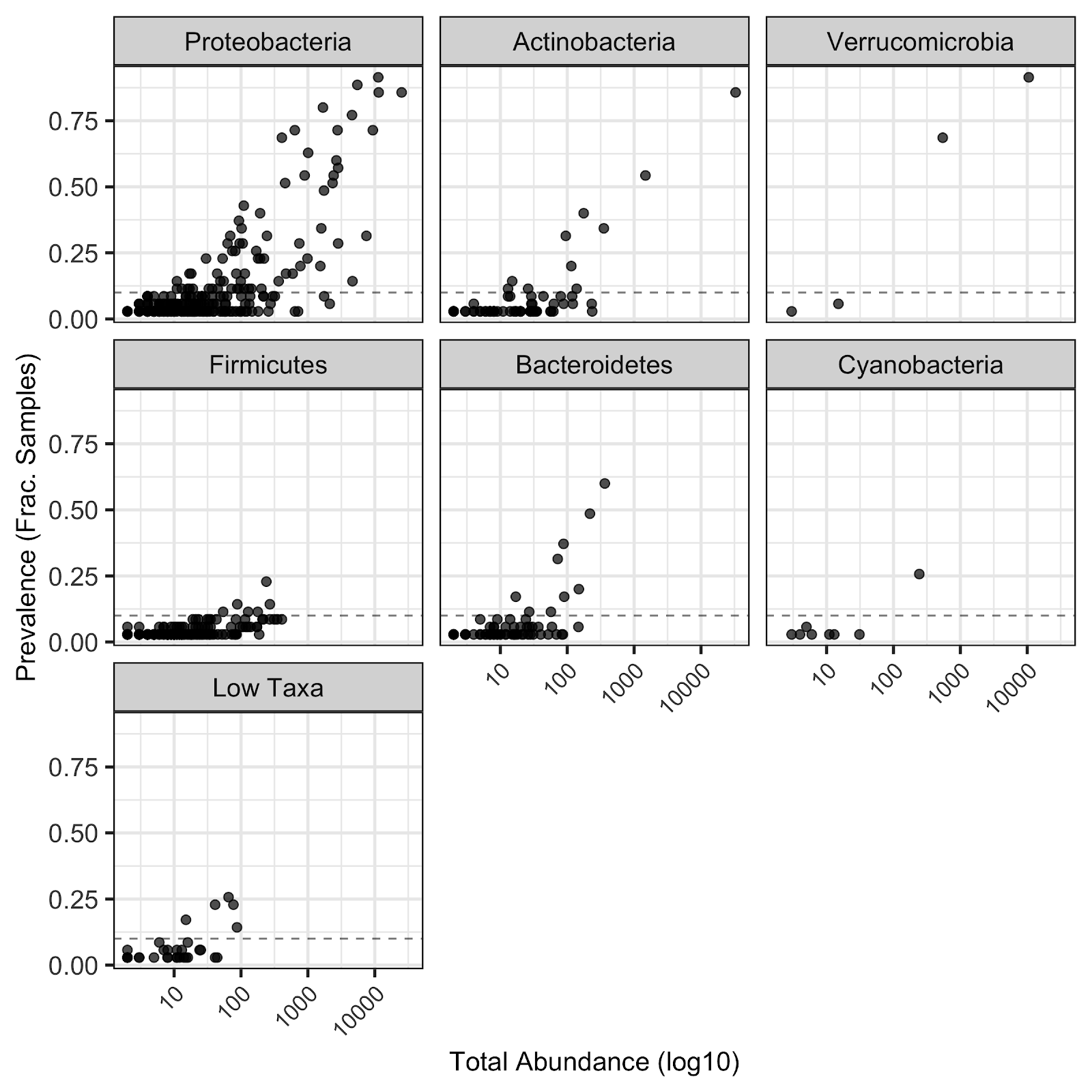
**

**Figure S2.** The Fraction of Prevalence (y - axis) versus Total abundance (x axis) shows a positive relationship: Proteobacteria (0.643), Actinobacteria (0.743), Verrucomicrobia (0.767), Firmicutes (0.614), Bacteroidetes (0.831), Cyanobacteria (0.983). Low taxa are the group of taxa with abundance lower than 1% of the total abundance.
