# Supplementary material for: Longitudinal variations in the gastrointestinal microbiome of the white shrimp, Litopenaeus vannamei
Source: PeerJ. 2021 Aug 2;9:e11827. doi: 10.7717/peerj.11827 (PMC8340905; doi:10.7717/peerj.11827)
Supplement: Supplemental Information 3 — The percentage of variation explained by PC1, PC2 and PC3 are indicated in the axis. Different colors represent different samples. Comparison of predicted microbial function among groups based on KEGG level-3. [file peerj-09-11827-s003.docx]

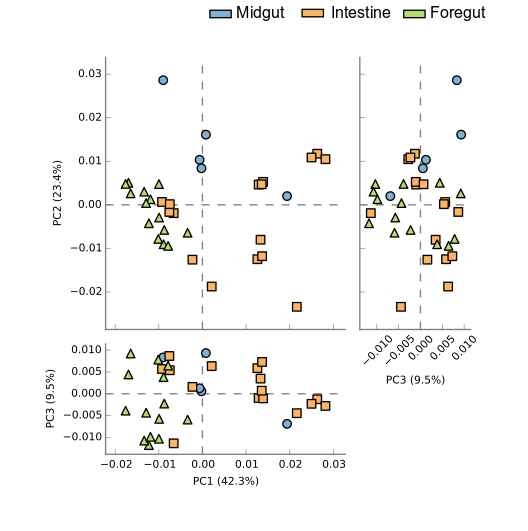


**Figure S4.** Principal component analysis (PCA) profile of predicted functions from digestive tract microbiota of white shrimp (Litopenaeus vannamei). The percentage of variation explained by PC1, PC2, and PC3 are indicated in the axis. Different colors represent different samples. Comparison of predicted microbial function among groups based on KEGG level-3.
